# Longitudinal Assessment of Muscle Involvement in Late‐Onset Pompe Disease Using Quantitative MRI: A Prospective Cohort Study

**DOI:** 10.1002/jcsm.70304

**Published:** 2026-06-01

**Authors:** Alice De Lorenzo, Johannes Forsting, Martijn Froeling, Anne‐Katrin Güttsches, Elena Enax‐Krumova, Felix Kleefeld, Menekse Öztürk, Ferdinand Knieling, Matthias Vorgerd, Robert Rehmann, Tobias Ruck, Lara Schlaffke

**Affiliations:** ^1^ Department of Neurology, BG‐University Hospital Bergmannsheil Ruhr‐University Bochum Bochum Germany; ^2^ Heimer Institute for Muscle Research BG‐University Hospital Bergmannsheil Bochum Germany; ^3^ Department of Radiology University Medical Centre Utrecht Utrecht the Netherlands; ^4^ Department of Pediatrics and Adolescent Medicine, University Hospital Erlangen Friedrich‐Alexander‐Universität (FAU) Erlangen‐Nürnberg Erlangen Germany; ^5^ Translational Pediatrics, Department of Pediatrics and Adolescent Medicine, University Hospital Erlangen Friedrich‐Alexander‐Universität (FAU) Erlangen‐Nürnberg Erlangen Germany; ^6^ Department of Neuropediatrics and Social Pediatrics, University Hospital of Pediatrics and Adolescent Medicine, St. Josef‐Hospital Ruhr‐University Bochum Bochum Germany; ^7^ Department of Information Technology Dortmund University of Applied Sciences and Arts Dortmund Germany

**Keywords:** diffusion tensor imaging, fat fraction, late‐onset Pompe disease, quantitative muscle MRI, water T2 relaxation time

## Abstract

**Background:**

Late‐onset Pompe disease (LOPD) is a progressive metabolic myopathy characterised by lysosomal glycogen accumulation and leading to secondary disruptions in autophagy and cellular energy metabolism. While enzyme replacement therapy (ERT) has improved outcomes, early detection remains critical, because irreversible muscle damage often precedes overt clinical symptoms. This study investigates whether quantitative MRI (qMRI) can detect subclinical disease progression and correlate with clinical decline.

**Methods:**

Over a 2‐year period, 25 participants (10 LOPD (disease duration 131 ± 127 months; ERT 76 ± 72 months; two dropouts) and 15 age/sex‐matched controls) underwent longitudinal clinical and MRI assessments at intervals of ~4 months (± 4 weeks; T1–T7). Clinical tests included strength measurements (Medical Research Council, MRC), the Quick Motor Function Measure (QMFM), patient‐reported outcomes (ACTIVLIM; Neuromuscular Symptom Score—NSS) and gait analysis. The 3.0T lower extremity MRI included multi‐echo, gradient‐echo, a Dixon‐based sequence for fat quantification (fat fraction [FF]), a multi‐echo, spin‐echo sequence for T2 mapping (wT2) and a spin‐echo EPI diffusion‐weighted sequence. Baseline differences in quantitative MRI (qMRI) metrics between patients and controls were assessed using multivariate analysis of variance. Longitudinal changes and associations with clinical outcomes were evaluated using linear mixed‐effects models (LMMs).

**Results:**

Group differences were found for ACTIVLIM (*p* ≤ 0.027), NSS (*p* ≤ 0.001) and MRC (*p* = 0,027) from T6/T7. Baseline differences of qMRI metrics between groups were found for FF (mean difference = 0.071, *p* < 0.001), wT2 (mean difference = 1.925, *p* < 0.001) and fractional anisotropy (mean difference = 0.010, *p* = 0.029). In muscles with FF < 10%, diffusion metrics did not differ (*p* ≥ 0.332), whereas wT2 remained elevated in LOPD (*p* < 0.001). LMMs revealed a significant increase in FF in LOPD patients from T4 onward (*p* ≤ 0.029), particularly in thigh muscles, while wT2 and diffusion metrics remained stable over time. LMMs demonstrated significant associations between thigh muscle qMRI metrics and clinical outcomes, for example between FF and QMFM (estimate: −5.16; *R* ^2^ = 0.76; *p* < 0.001) or wT2 and QMFM (estimate: −0.64; *R* ^2^ = 0.83; *p* = 0.009). Interestingly, wT2 showed a modest positive association over time for ACTIVLIM (estimate: 0.005; *R* ^2^ = 0.517; *p* = 0.010).

**Conclusion:**

Although muscle function is measurable clinically, qMRI captured alterations beyond clinical detection; its correlation with clinical scores supports qMRI as a more sensitive surrogate marker. Our findings indicate that FF changes preceded alterations in clinical outcome, highlighting sensitivity to early disease progression. The lack of a consistent temporal pattern in wT2 and diffusion metrics warrants further studies to fully understand underlying mechanisms in LOPD. Larger, long‐term studies are needed to validate qMRI for monitoring disease progression and therapeutic response in LOPD.

**Abbreviations**
6‐MWD: 6‐min walking distance
10‐MWT: 10‐m walk test
EPG: extended phase graph
ERT: enzyme replacement therapy
FA: fractional anisotropy
FF: fat fraction
FOV: field of view
GAA: lysosomal acid alpha‐glucosidase
IDEAL: iterative decomposition of water and fat with echo asymmetry and least‐squares estimation
iWLLS: iterative weighted linear least squares
λ1: axial diffusivity
LMM: linear mixed‐effects model
LOPD: late‐onset Pompe disease
MANOVA: multivariate analysis of variance
MD: mean diffusivity
mDTI: muscle diffusion tensor imaging
MRC: Medical Research Council
MSOT: multispectral optoacoustic tomography
NMD: neuromuscular disease
NSS: neuromuscular symptom score
PCA: principal component analysis
QMFM: quick motor function measure
qMRI: quantitative magnetic resonance imaging
RD: radial diffusivity
SNR: signal‐to‐noise ratio

## 1 Introduction

Late‐onset Pompe disease (LOPD) is a rare autosomal recessive metabolic myopathy caused by mutations in the *GAA* gene, resulting in deficient activity of lysosomal acid alpha‐glucosidase (GAA) [1]. This enzyme deficiency leads to progressive intralysosomal glycogen accumulation, predominantly affecting skeletal, cardiac and smooth muscles. Beyond glycogen storage, the pathophysiology involves secondary disruptions in autophagy and energy metabolism, contributing to oxidative stress, mitochondrial dysfunction and intracellular toxicity [2]. Diagnosis is established by biochemical assessment of GAA activity, preferably via dried blood spot testing, followed by *GAA* gene sequencing [3]. Clinical onset typically occurs in adolescence or adulthood, presenting with slowly progressive, symmetric proximal muscle weakness, particularly in the pelvic and shoulder girdles. Diaphragmatic involvement is common and often precedes motor symptoms, contributing to progressive ventilatory insufficiency in up to 80% of patients [4].

Enzyme replacement therapy (ERT) with recombinant human GAA, introduced in 2006, marked a turning point in the management of LOPD, though skeletal muscle response remains limited due to suboptimal cellular uptake and autophagic build‐up [5, 6]. Second‐generation ERTs—such as avalglucosidase alfa and cipaglucosidase alfa—show improved muscle targeting and enhanced functional benefits [7, 8]. Meanwhile, adeno‐associated virus (AAV)–based gene therapy is under investigation, showing encouraging preclinical results, though challenges with immunogenicity, hepatotoxicity and dosing remain [9].

While current and emerging therapies are promising for improving outcomes, their success depends on early initiation, ideally before irreversible muscle damage becomes clinically apparent [10]. In this context, advanced imaging modalities offer an innovative approach of detecting structural abnormalities at a subclinical stage—before they become apparent through routine clinical examination. Techniques such as multispectral optoacoustic tomography (MSOT), which can detect glycogen, collagen and lipid changes with greater sensitivity than current methods, are still in the early stages of clinical translation [11]. In contrast, MRI techniques are already routinely used to visualise patterns of muscle involvement in the diagnostic workup of neuromuscular disorders, with quantitative MRI methods offering superior diagnostic value compared with purely qualitative image assessment [12].

Quantitative muscle MRI (qMRI), including Dixon‐based fat quantification, T2 mapping and diffusion tensor imaging (DTI), is gaining recognition as a noninvasive surrogate biomarker in LOPD [13, 14, 15, 16, 17]. These techniques detect microstructural changes, such as fat replacement, early oedema and altered fibre orientation, prior to overt changes on conventional MRI [13, 14, 15, 16, 17]. Supporting these findings, Rohm et al. demonstrated diffusion alterations in a presymptomatic mouse model of Pompe disease in the absence of fat replacement. These changes showed a positive correlation with autophagic markers and muscle fibre diameter, aligning with the pathophysiology of Pompe disease, in which impaired autophagy plays a central role [18]. However, comparably detailed longitudinal clinical and MRI assessments in patients with LOPD are still lacking.

Therefore, this study aimed to investigate whether clinical progression in LOPD correlates with qMRI changes, and whether qMRI can detect slow, subclinical disease progression that remains undetected by routine examination, a well‐recognised challenge in slowly progressive degenerative disorders that underscores the need for sensitive biomarkers.

## 2 Methods

### 2.1 Study Population

Initially, 10 patients with genetically confirmed LOPD and 15 healthy controls were enrolled in the study. To reduce confounding and strengthen group comparisons, the controls were matched for final analysis to the patient group in terms of age and sex. Inclusion criteria comprised MRI compatibility, age > 18 years and voluntary participation. Exclusion criteria for healthy volunteers were a medical history of neuromuscular diseases (NMDs) and injuries in the lower extremity within 12 months before study enrolment. All participants provided written informed consent. Clinical assessments and MRI scans were conducted approximately every 4 months (± 4 weeks) over a 2‐year period, allowing for longitudinal monitoring and analysis. Each combined clinical and imaging visit was defined as a study timepoint (T1–T7), with T1 representing the baseline assessment at study entry and T7 the final evaluation at 24 months, while T2–T6 denoted the intermediate follow‐up visits. The study was approved by the local ethics committee (approval number 15‐5281).

### 2.2 Clinical Assessments

Clinical assessment included muscle strength evaluation (Medical Research Council [MRC] scale), standardised questionnaires (Quick Motor Function Measure [QMFM], Neuromuscular Symptom Score [NSS], ACTIVLIM) and gait analysis (6‐min walking distance [6‐MWD], 10 m walking test [10MWT]) [19, 20, 21]. The MRC scale was used to qualitatively grade the strength (0–5) in key muscle groups, while dynamometry provided quantitative measurement of lower limb force during flexion and extension. QMFM, NSS and ACTIVLIM assessed motor function and daily activity limitations. Gait tests combined time‐based and functional performance metrics.

### 2.3 MRI Acquisition and Sequences

Following the protocol used by Forsting et al. [22], participants were scanned in a supine, feet‐first position using a Philips 3.0T Achieva system with a 16‐channel Torso XL coil. Lower limb imaging included the thighs, split into two overlapping FOVs (480 × 276 × 150 mm^3^) and the lower legs, imaged with a single FOV (single FOV of the same dimensions, whose upper edge was positioned 60 mm distal to the tibial plateau), with planes oriented perpendicular to the femur and tibia. The protocol, following Schlaffke et al. [23], comprised three main sequences: Dixon 4‐point sequence with voxel resolution 1.5 × 1.5 × 6.0 mm^3^; TR/TE 210/2.6, 3.36, 4.12, 4.88 ms; flip angle 8°; SENSE: 2; multi‐echo spin‐echo (MESE) for water quantification with 17 echoes; Cartesian k‐space readout; voxel size 3.0 × 3.0 × 6.0 mm^3^; TR/TE 4598/17 × Δ7.6 ms; excitation/refocusing flip angles 90°/180°; SENSE: 2 and diffusion‐weighted spin‐echo EPI with voxel size 3.0 × 3.0 × 6.0 mm^3^; TR/TE 5000/57 ms; SPAIR/SPIR fat suppression; SENSE: 1.9; 42 diffusion directions across eight different b‐values (0–600). A separate noise measurement was additionally obtained, applying the DWI settings but without gradient or RF excitation (only receiver channels active). The entire session lasted approximately 36 min.

### 2.4 Data Preprocessing

Data processing followed the same methodological pipeline as in the study by Forsting et al. [22]. Data were preprocessed using QMRITools in accordance with established methods [23, 24]. Diffusion data were denoised via PCA [25] and registered separately for each leg to correct for motion and eddy currents. Tensor metrics were computed using intravoxel incoherent motion (IVIM) modelling and an iterative weighted linear least squares (iWLLS) algorithm [26, 27, 28]. Dixon data were processed with the IDEAL method (single T2* assumption) to generate water and fat maps [29], which served as the basis for manual segmentation. T2 mapping data were analysed using extended phase graph (EPG) fitting [30].

### 2.5 Muscle Segmentation

Muscle analysis was conducted following the segmentation approach described by Forsting et al. [22]. Twelve thigh muscles (vastus lateralis, vastus medialis, vastus intermedius, rectus femoris, sartorius, semimembranosus, semitendinosus, biceps femoris—both heads, adductor magnus, adductor longus, adductor brevis and gracilis) and 11 lower leg muscles (tibialis anterior, extensor digitorum longus, extensor hallucis longus, flexor digitorum longus, flexor hallucis longus, gastrocnemius—caput medialis and lateralis, soleus, fibularis longus and tibialis posterior) were segmented on Dixon water images. Initial segmentation was performed using the automated segmentation function in QMRITools [24], and subsequently refined manually for both lower limbs by a trained examiner with knowledge of lower limb muscle anatomy, under the supervision of an experienced colleague (JF, 8 years), following a standardised and previously established quantitative muscle MRI segmentation protocol. For further analysis, individual muscles were grouped into anatomically and functionally related muscle groups to facilitate direct correlations between quantitative MRI metric and clinical motor function measures and to better reflect the overall pattern of muscle involvement. The vastus lateralis, vastus intermedius, vastus medialis, rectus femoris and sartorius were combined to form the group of thigh extensors. The biceps femoris (long and short head), semimembranosus and semitendinosus were grouped as thigh flexors. The adductor longus, adductor brevis and gracilis were analysed together as thigh adductors, while the tibialis anterior, extensor digitorum longus and extensor hallucis longus were classified as lower leg extensors and the fibularis longus, flexor digitorum longus, flexor hallucis longus, gastrocnemius medialis, gastrocnemius lateralis, soleus and tibialis posterior were classified as lower leg flexors. For each muscle group, compound scores were calculated based on the summed muscle volumes, enabling standardised group‐level quantitative analysis.

### 2.6 Statistical Analysis

To examine baseline differences in qMRI metrics between the patient and control group, a multivariate analysis of variance (MANOVA) was employed with group (patient vs. control), body side and muscle group as fixed factors. Muscles were clustered in the following muscle groups: knee flexors (biceps femoris, semitendinosus, semimembranosus), knee extensors (rectus femoris, sartorius, vastus medialis, vastus lateralis), adductors (adductor magnus, adductor longus, adductor brevis, gracilis) in the thigh, dorsiflexors (extensor digitorum longus, extensor hallucis longus, tibialis anterior) and plantarflexors (fibularis longus, flexor digitorum longus, flexor hallucis longus, gastrocnemius medialis, gastrocnemius lateralis, soleus, tibialis posterior) in the leg. Post hoc *t*‐tests were conducted for each muscle group using Tukey's HSD test following the univariate ANOVA results, adjusting for multiple comparisons. For diffusion metrics analysis, muscles with an SNR < 10 were excluded due to poor data quality [31, 32]. In a subsequent cross‐sectional analysis, muscles with fat fraction (FF) > 10% were excluded to compare water T2 relaxation times and diffusion metrics between low‐fat muscles of patients and controls at baseline [14].

Longitudinal changes in qMRI metrics were assessed with linear mixed‐effects models (LMMs) fitted using restricted maximum likelihood estimation (REML). Fixed effects included qMRI metric at baseline, session, diagnostic group and muscle group, with body side, BMI and age added as covariates. To capture differential progression patterns across subgroups, interaction terms between time, group and muscle groups were included. Random intercepts were specified at two hierarchical levels—subject and muscle nested within subject—to account for repeated measurements and intra‐individual variability across muscles. To account for temporal autocorrelation within subjects, a continuous autoregressive correlation structure of order 1 (CAR [1]) was applied across repeated time points for each subject‐muscle‐side combination. A comparable modeling strategy was applied to the clinical outcome measures.

To relate MRI to clinical outcomes, compound scores were calculated for all thigh and leg muscles, weighted by each muscle's segmentation mask volume. Associations between the clinical outcome and qMRI metrics over time were examined with LMMs including fixed effects for qMRI metric, time and their interaction. Random intercepts and slopes for time were included at the subject level to account for individual variability.

In a secondary exploratory analysis, we examined the influence of wT2, FA and MD on changes in FF over the entire study period using a linear regression model. Standard errors were made cluster‐robust to account for repeated measures within subjects.

All statistical analyses were conducted using R (version 4.5.1., R Foundation for Statistical Computing, Vienna, Austria), with a significance level of *p* < 0.05.

## 3 Results

### 3.1 Clinical Characteristics of Patients

**FIGURE 1:**
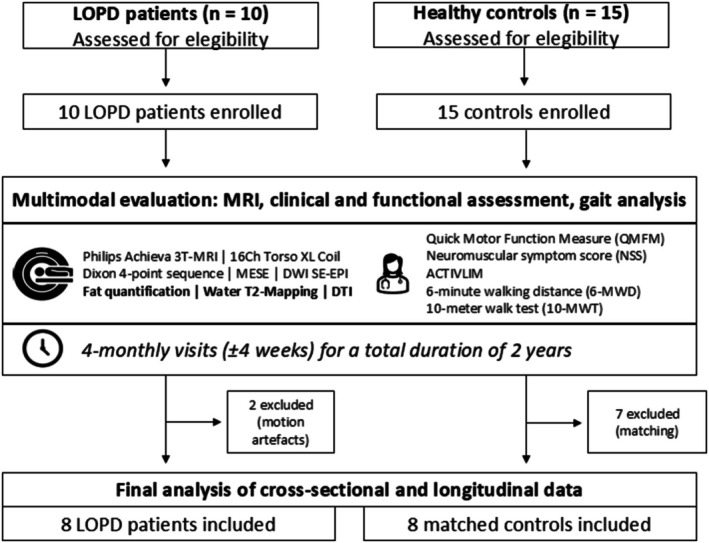
Flowchart of study procedure.

Our cohort included at first 10 patients with genetically confirmed LOPD and 15 healthy controls. Two LOPD patients were excluded due to corrupted MRI data; therefore, the final analysis comprised eight LOPD patients (four female; age range: 18–75 years; mean age: 42.0 ± 20.38 years; BMI: 22.0 ± 2.88) and eight matched controls (four female; age range: 22–65 years; mean age: 39.75 ± 18.06 years; BMI: 23.91 ± 2.89). The mean disease duration at baseline in the study cohort was 131 ± 126.85 months (range 4–391 months), and the mean time since manifestation was 212 ± 121.96 months (see Table 1). All patients received ERT with a mean treatment duration of 76 ± 71.91 months. QMFM results ranged from 33 to 64 (mean 49 ± 13.05), while ACTIVLIM ranged from 26 to 20 (mean 22 ± 1.98), and NSS from 21 to 42 (mean 35 ± 6.97). Due to corrupt MRI data in two LOPD patients, only eight LOPD patients and matched controls were included in the final analysis (see Figure 1).

**TABLE 1:** Demographic, clinical data and clinical test of patients with late‐onset Pompe disease (LOPD).

Code	Age (years)	Height (cm)	BMI	Sex	Months since manifestation	Months since diagnosis	Duration of ERT (months)	QMFM	ACTIVLIM	NSS	MRC mean legs	6MWT	10m_Time
Patient 1	47	193	19.87	m	184	155	153	33	23	30	4.31	422.5	7.31
Patient 2	45	188	18.39	m	487	391	187	34	24	36	4.13	426.0	7.25
Patient 3	66	183	20.31	m	93	16	11	40	21	21	4.44	542.0	5.94
Patient 4	34	187	26.31	m	138	94	81	59	20	42	4.88	495.0	8.84
Patient 5	22	177	22.02	w	222	222	126	64	21	40	5.00	528.0	6.00
Patient 6	29	169	26.26	w	143	96	10	59	21	40	4.75	472.0	5.11
Patient 7	18	175	21.22	w	174	66	39	62	22	37	4.88	480.0	6.84
Patient 8	75	163	22.58	w	254	4	2	43	26	31	3.13	320.0	11.40
Patient 9*	50	165	25.71	w	204	108	84	—	—	—	—	—	—
Patient 10*	58	177	28.09	m	13	1	8	—	—	—	—	—	—
Mean ± STD**	42 ± 20.38	179 ± 10.09	22 ± 2.88	—	212 ± 121.96	131 ± 126.85	76 ± 71.91	49 ± 13.05	22 ± 1.98	35 ± 6.97	4 ± 0.61	461 ± 70.99	7 ± 1.99

*Note:* * = were excluded in the analysis; ** = mean and standard deviation of the eight included participants.

**FIGURE 2:**
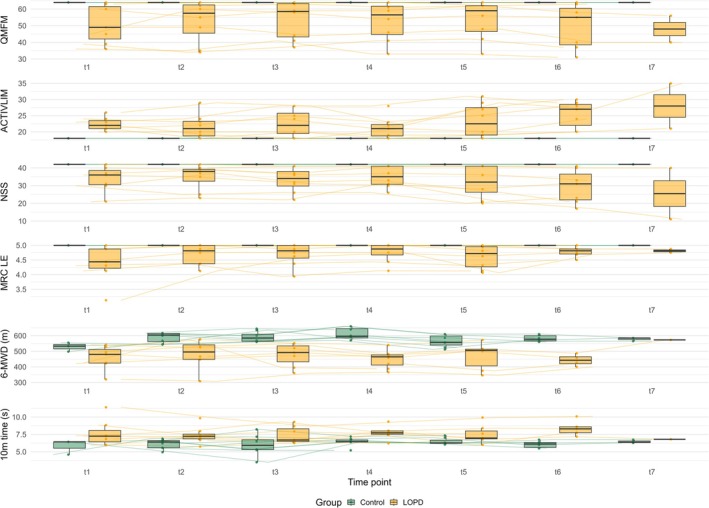
Longitudinal trajectories of clinical outcome measures in individuals with late‐onset Pompe disease (LOPD) compared with healthy controls. 6‐MWD, 6‐min walking distance; MRC LE, MRC sum score of lower extremity; NSS, Neuromuscular Symptom Score; QMFM, Quick Motor Function Measure.

LMMs did not show significant longitudinal differences between groups for QMFM, 6MWD and 10‐Meter Walking Time (see Figure 2). In contrast, group differences in ACTIVLIM score emerged at time point T6 (*p* ≤ 0.027), with significant intra‐group progression observed in the LOPD group from T6 onwards (*p* ≤ 0.008). The same pattern was observed for the NSS and MRC sum score, where intergroup differences became significant at T7 (NSS: *p* = 0.001; MRC sum score: *p* = 0.027), coinciding with progressive worsening in the LOPD group from T6 onwards (NSS: *p* ≤ 0.046; MRC sum score: *p* ≤ 0.016).

### 3.2 MRI Findings

**FIGURE 3:**
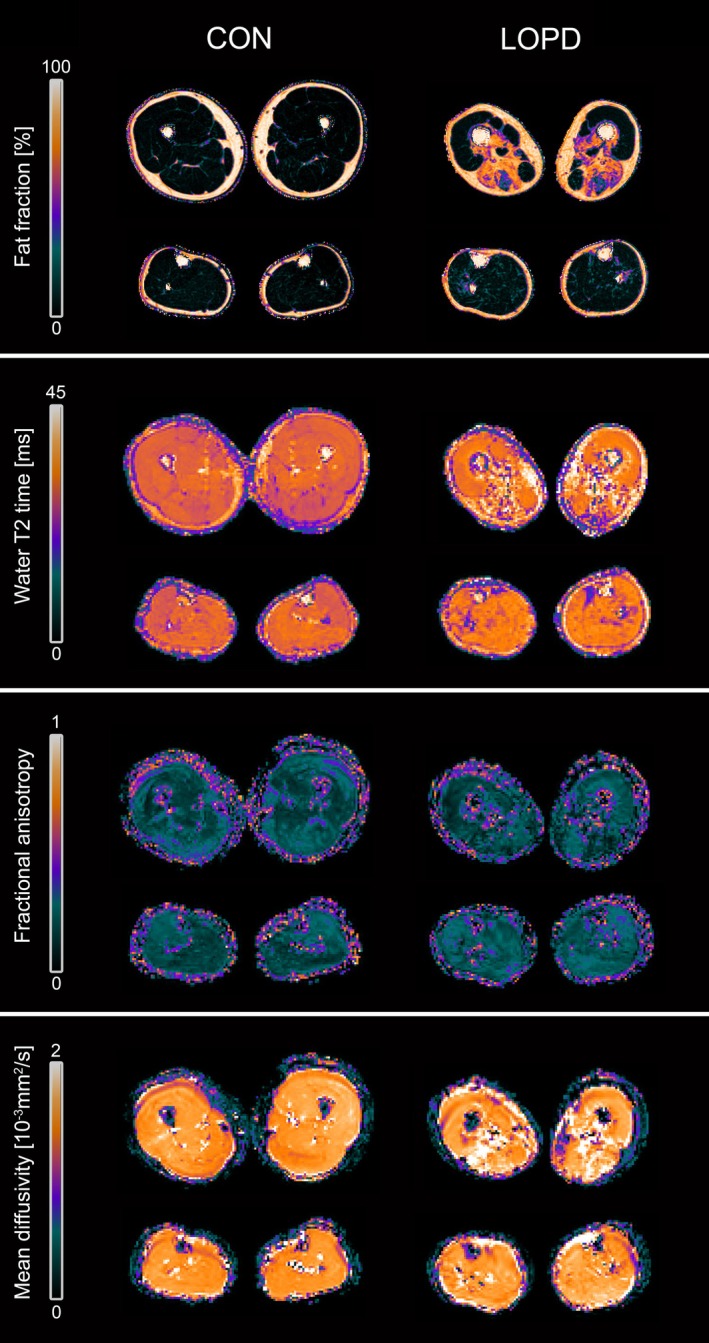
Overview of parameter maps for fat fraction (FF), water T2 relaxation time (wT2), fractional anisotropy (FA) and mean diffusivity (MD) of a representative late‐onset Pompe disease patient (LOPD) and healthy control (CON).

Due to a technical malfunction during image acquisition, a substantial portion of MRI datasets at time point T1 was corrupted and subsequently excluded from analysis. As a result, time point T2 was designated as the baseline for evaluating the MRI data. Additionally, scans from two LOPD patients exhibited pronounced motion artefacts, necessitating their exclusion from the dataset. In total, 83 MRI datasets were deemed suitable and included in the final analysis. Figure 3 gives an exemplary overview of parameter maps of qMRI metrics in a representative patient and healthy control.

#### 3.2.1 Differences of qMRI Metrics Between Patient and Control Group at Baseline

**FIGURE 4:**
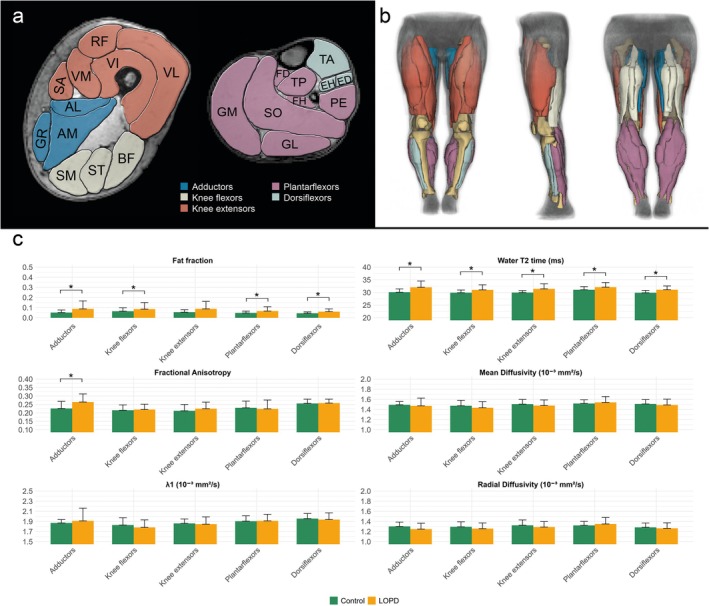
Overview of segmented muscles and corresponding muscle groups in a representative participant (a—cross‐sectional; b—front, side, and back view). Bar plots show mean qMRI metrics for patients with LOPD and control group (c). The lines show the standard deviation. *adjusted *p* < 0.05. AB, adductor brevis; AL, adductor longus; AM, adductor magnus; BF, biceps femoris; ED, extensor digitorum longus; EH, extensor hallucis longus; FD, flexor digitorum longus; FH, flexor hallucis longus; FL, fibularis longus; GM, gastrocnemius medialis; GL, gastrocnemius lateralis; GR, gracilis; PE, peroneal lodge; RF, rectus femoris; SA, sartorius; SO, soleus; SM, semimembranosus; ST, semitendinosus; TA, tibialis anterior; TP, tibialis posterior; VI, vastus intermedius; VL, vastus lateralis; VM, vastus medialis.

In the cross‐sectional comparison, FF was significantly higher in LOPD patients across all muscle groups (mean difference = 0.071 [95% CI: 0.039–0.103], *p* < 0.001) except the knee extensors (*p* = 0.051; see Figure 4). wT2 values were also consistently elevated in LOPD (mean difference = 1.925 [95% CI: 1.481–2.369], *p* < 0.001). Among diffusion tensor imaging (DTI) metrics, only fractional anisotropy (FA) differed significantly between groups (mean difference = 0.010 [95% CI: 0.001–0.020], *p* = 0.029), which, according to post hoc tests, was driven by the adductors (mean difference = 0.039 [95% CI: 0.013–0.066], *p* = 0.005). No main effects were found for MD (*p* = 0.403), L1 (*p* = 0.191), or RD (*p* = 0.733). In muscles with FF < 10%, diffusion parameters did not differ (*p* ≥ 0.332), whereas wT2 remained elevated in LOPD (*p* < 0.001), except in the knee flexors (*p* = 0.088; see Figure S1).

#### 3.2.2 Longitudinal Changes of qMRI Metrics

**FIGURE 5:**
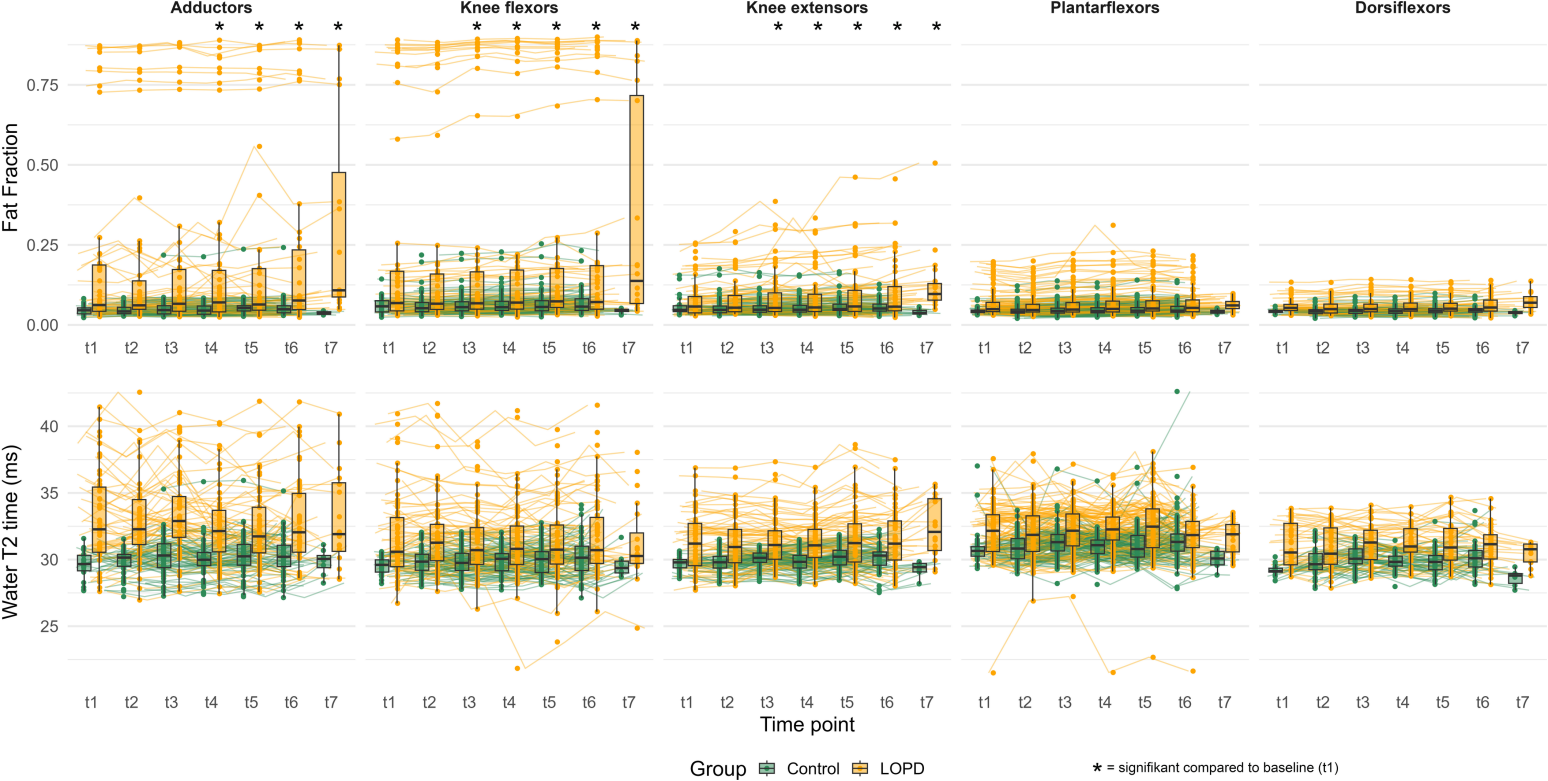
Longitudinal trajectories of the qMRI metrics fat fraction and water T2 relaxation time in individuals with LOPD compared with healthy controls. *adjusted *p* < 0.05.

Compared with the baseline, a statistically significant divergence in the longitudinal trajectories of the two groups was observed for FF from T4 onward (*p* ≤ 0.029; see Figure 5). In the LOPD group, a significant increase in FF was detectable from T3 when considering all muscle groups (*p* < 0.001). Post hoc analyses revealed that this effect was driven by thigh muscles (adductors: from T4, *p* ≤ 0.020; knee extensors: from T3, *p* ≤ 0.001; knee flexors: from T3, *p* ≤ 0.002).

Although overall group divergences were observed at T5 (*p* = 0.003) when considering all muscles, post hoc analyses did not confirm statistically significant wT2 changes from baseline within the patient group.

Diffusion metrics did not show any significant differences in longitudinal trajectories of both groups throughout the study period (FA: *p* ≥ 0.176; MD: *p* ≥ 0.121; L1: *p* ≥ 0.196; RD: *p* ≥ 0.068).

In the subgroup of muscles with FF below 10%, wT2 remained significantly different between groups at individual time points (T3: *p* = 0.03). However, post hoc tests did not reveal a consistent temporal pattern in the evolution of qMRI parameters. Diffusion metrics remained stable and showed no group differences over time (FA: *p* ≥ 0.226; MD: *p* ≥ 0.296; L1: *p* ≥ 0.211; RD: *p* ≥ 0.171).

#### 3.2.3 Correlations of the Longitudinal Evaluation of qMRI Metrics and Clinical Examinations

**FIGURE 6:**
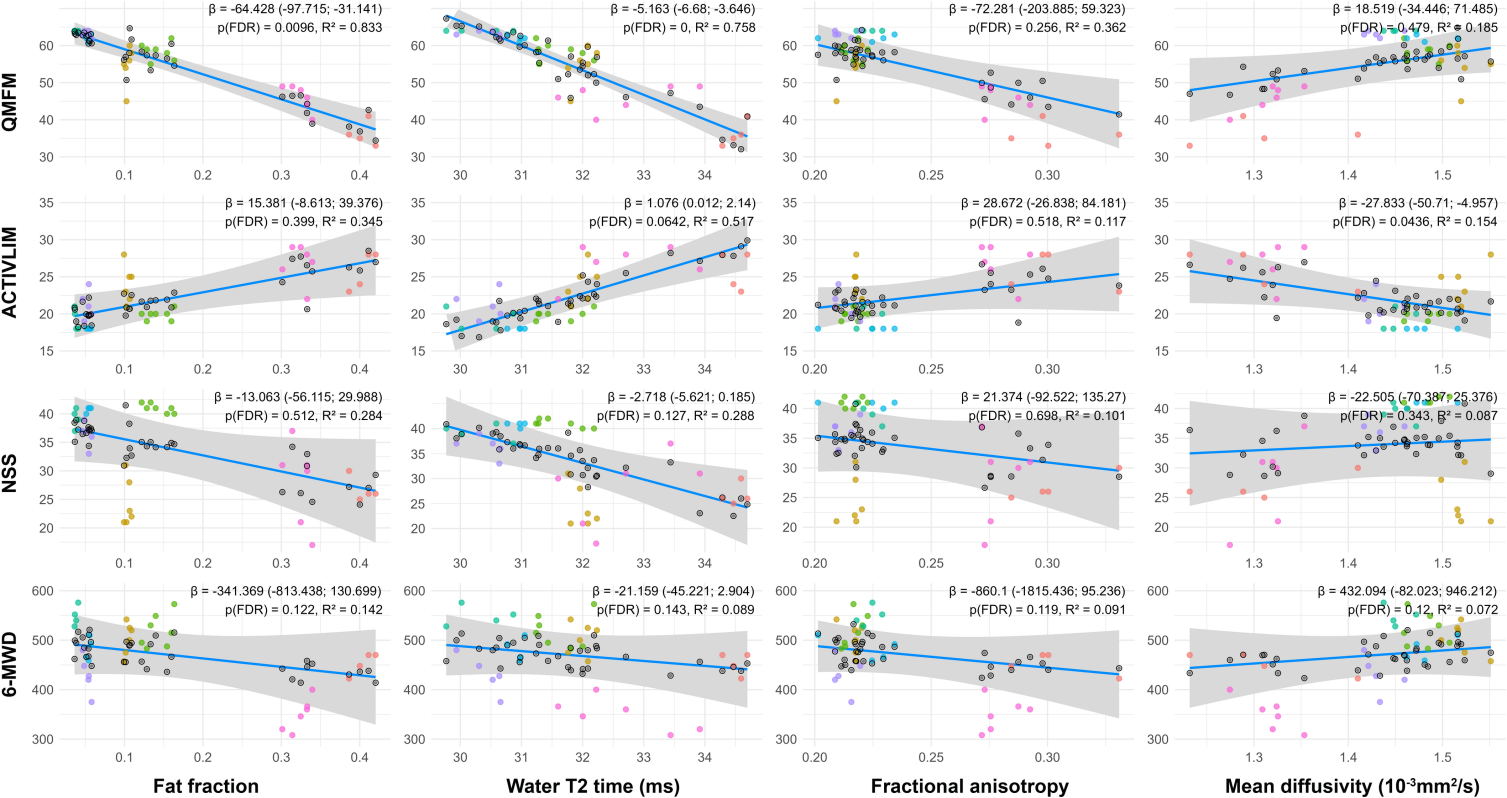
Correlations between thigh muscle qMRI metrics and selected clinical outcomes in patients with LOPD. Each color represents an individual subject at different time points, grey points represent modeled data. Beta estimates (*β*), 95% confidence intervals, FDR‐adjusted *p*‐values and marginal *R* ^2^ values are shown for each association. 6‐MWD, 6‐min walking distance; NSS, Neuromuscular Symptom Score; QMFM, Quick Motor Function Measure.

LMMs revealed significant associations between thigh muscle qMRI parameters and clinical outcomes (Figure 6). Specifically, FF of the thigh muscles was negatively associated with QMFM (estimate: −5.16 [95% CI: −6.68; −3.65]; *R* ^2^ = 0.76; *p* < 0.001), as was water T2 (wT2; estimate: −0.64 [95% CI: −0.98; −0.31]; *R* ^2^ = 0.83; *p* = 0.009). Thigh muscle wT2 also negatively influenced the MRC sum score (estimate: −0.25 [95% CI: −0.41; −0.09]; *R* ^2^ = 0.55; *p* = 0.014). In addition, MD (estimate: −27.8 [95% CI: −50.7; −5.0]; *R* ^2^ = 0.15; *p* = 0.044) and RD (estimate: −25.8 [95% CI: −46.8; −4.8]; *R* ^2^ = 0.17; *p* = 0.042) were negatively associated with ACTIVLIM.

Furthermore, significant longitudinal associations were observed between several qMRI parameters and clinical outcomes over time (Figure S2). RD (estimate: 0.103 [95% CI: 0.039; 0.166]; *R* ^2^ = 0.090; *p* = 0.006), λ1 (estimate: 0.097 [95% CI: 0.027; 0.167]; *R* ^2^ = 0.113; *p* = 0.0173) and MD (estimate: 0.104 [95% CI: 0.037; 0.171]; *R* ^2^ = 0.087; *p* = 0.008) were positively associated with NSS over time. For ACTIVLIM, wT2 exhibited a modest positive association over time (estimate: 0.005 [95% CI: 0.002; 0.008]; *R* ^2^ = 0.517; *p* = 0.0102).

#### 3.2.4 Influence of wT2 and Diffusion Metrics on FF Change Over Study Duration

wT2 at baseline was significantly associated with the FF change over study duration (*β* = 0.004 [95% CI: 0.00322; 0.00434], *p* = 0.009; see Figure S3), while FA and MD at baseline were not significantly associated (*p* ≥ 0.596).

## 4 Discussion

By combining repeated quantitative imaging with serial clinical assessments, this study was designed to analyse sensitivity of MRI parameters for detecting subtle within‐patient changes compared with clinical outcomes and to identify qMRI measures with potential utility as surrogate markers for disease activity and treatment response.

Functional outcome measures (QMFM, 6‐MWD and 10‐MWT) revealed stable group‐level trajectories over the 2‐year observation period in patients receiving ERT. The only objective parameter that showed a worsening was the MRC sum score, approximately after the 2‐year observation (T7). Notably, a modest improvement in QMFM at T2 was observed, an unexpected finding in a progressive NMD. This change may reflect intraindividual variability or transient clinical improvement rather than sustained disease reversal. Previous studies have shown an early beneficial response to ERT in most patients followed by heterogeneous gains and declines, and have proposed explanations such as renewed motivation, improved adherence or recent ERT administration for temporary performance gains [33, 34]. Given that most patients had been on long‐term ERT and that assessments were typically performed on the day of ERT infusion, these applications only partly apply in our cohort. In contrast to QMFM, ACTIVLIM and NSS displayed a coherent temporal pattern, with measurable deterioration after approximately 20–24 months (T6 for ACTIVLIM and T7 for NSS) in the LOPD group. These findings are noteworthy considering current clinical trial practices. Large recent trials in Pompe disease, such as PROPEL and COMET [7, 8], have relied primarily on forced vital capacity (FVC) and 6‐MWD as primary endpoints. Our data suggest that patient‐reported outcome measures can identify longitudinal change and could serve as complementary clinical endpoints in future trials.

Regarding the MRI parameters, the most robust and consistent imaging findings were observed for FF, which increased significantly over time in the LOPD group from 8 months onward (T3). While Vaeggemose et al. did not find qMRI changes over 8 months and reported no correlations with clinical parameters, various studies reported average FF increases of 0.9 to 1.8% per year and good correlation with clinical findings [15, 18, 35, 36]. Possible explanations for these discrepant findings include small sample size, shorter follow‐up, different muscle selection, lower baseline disease burden or differing MRI acquisition and analysis pipelines. Our study contributes the important finding that significant changes can be observed as early as after 8 months in a small, heterogeneous cohort of LOPD patients. Importantly, FF increased prior to any significant changes in clinical scores, suggesting that it is an early and sensitive biomarker of disease progression. Because FF in thigh muscles correlated highly with clinical outcome measures, these findings highlight FF as a potential surrogate endpoint in future trials. The longitudinal FF changes in our LOPD cohort exceeded the random fluctuations seen in healthy controls, which accounted for FF changes of 0.43% in an overlapping cohort acquired and processed using the same pipeline [22]. This underlines that the observed FF progression reflects true pathological change rather than measurement variability. Importantly, no relevant changes were observed in the lower leg muscles, which supports focusing future longitudinal MRI studies in LOPD on thigh musculature alone to reduce scan time without loss of sensitivity. Moreover, significant FF changes were detected despite scanning only a partial thigh segment, suggesting that full‐volume coverage may not necessarily be required.

However, it must be considered that FF reflects irreversible replacement of muscle by fat; once established, these changes cannot be reversed by therapeutic interventions. Thus, although FF is highly sensitive for detecting disease progression and can identify early tissue loss before clinical decline, it primarily reflects permanent rather than ongoing damage, modifiable disease activity [12]. Therefore, complementary markers that detect pathophysiological changes preceding fat replacement, for example other quantitative MRI parameters, are needed.

wT2 has been proposed as such a potential marker of subclinical damage in muscles of still‐normal appearance in LOPD, because muscles with higher wT2 values underwent faster fat replacement [13, 37]. In our cohort, wT2 was consistently higher in LOPD patients than in controls, but no consistent longitudinal change was detectable at the group level. One explanation is heterogeneity of individual disease trajectories: wT2 may increase in some participants and decrease in others, for example, artifactually as fat replacement increases, resulting in an absence of consistent trends [17, 38]. Conversely, elevated wT2 values are not specific and can reflect ongoing pathological processes such as muscle oedema, inflammation, autophagic or lysosomal stress that do not necessarily result in linear progression [39]. Nonetheless, the good cross‐sectional correlation of wT2 with clinical measures and its association with greater decline in ACTIVLIM over time indicate that wT2 can add complementary value to FF measurements by identifying potentially modifiable pathology that FF alone does not capture. In an exploratory secondary analysis, it could be shown that higher baseline wT2 was associated with a higher increase of FF (0.2% FF per 1 ms wT2 per year), suggesting that elevated wT2 predicts faster fat replacement in LOPD muscles. This observation aligns with previous findings demonstrating a greater progression tendency in muscles with pathological T2 values [13]. However, while FF showed a consistent and progressive increase over time, water T2, analysed using the same statistical approach, displayed a more variable temporal behaviour, likely reflecting the dynamic and fluctuating nature of disease activity, which may explain the absence of robust longitudinal changes at the group level.

Another promising candidate in detecting muscle changes in non‐fat‐replaced muscles of LOPD is diffusion MRI [40]. Cross‐sectional studies to date, however, report heterogeneous results. In non‐fat‐replaced muscles, reductions in MD, λ1 and RD have been interpreted as indicative of intracellular debris accumulation and lysosomal enlargement, whereas increases in FA in the absence of MD changes have been linked with fibre atrophy [14, 17]. Direct comparisons between studies are limited by differences in acquisition protocols and processing pipelines. Interestingly, preclinical longitudinal work in a Pompe mouse model linked reductions in MD and RD to p62 accumulation as a marker for autophagic buildup, supporting a mechanistic basis for diffusion changes; however, translational caution is warranted because the mouse model lacks inflammatory changes and fat replacement seen in humans [18]. In our cohort, no significant longitudinal changes of diffusion metrics were observed, which may reflect the more complex pathophysiology in human LOPD, shorter relative study intervals compared with the compressed disease timeline in mice and the necessity to exclude DTI datasets affected by advanced fat replacement in some participants. Although our cohort's heterogeneity improves generalisability, validation of diffusion metrics as early biomarkers will require larger, more homogenous samples and ideally long‐term follow‐up of therapy‐naïve, presymptomatic carriers with harmonised acquisition and postprocessing. Nevertheless, cross‐sectional correlations to clinical outcomes and associations of MD and RD with greater NSS decline over time suggest diffusion metrics remain a biologically plausible and potentially valuable adjunct to FF that merits further longitudinal investigation. The absence of a consistent temporal pattern in wT2 and diffusion measures could reflect the dynamic balance between transient intracellular oedema, autophagic stress and compensatory repair processes. It is conceivable that wT2 elevations mark fluctuating metabolic stress rather than structural damage, whereas diffusion changes may indicate microstructural reorganisation preceding irreversible degeneration [13]. These nuances highlight the need for combined multiparametric MRI approaches and integration with molecular biomarkers.

This study has several limitations. The cohort was heterogeneous for disease stage, age and function, increasing outcome variability and potentially diluting group‐level effects. A software‐related reconstruction issue affected the initial MRI acquisitions, resulting in incomplete Dixon datasets at the first time point, while all subsequent scans were acquired without this limitation. Changes and correlations were anticipated within the 2‐year timespan given the typically slow, progressive nature of the disease; however, the absence of detectable early markers suggests that longer follow‐up durations may be necessary to capture such dynamics reliably. Technical issues at T1 and motion artefacts required exclusion of a substantial proportion of MRI datasets, which represents a potential source of bias in the longitudinal evaluation. This led to the need for advanced statistical methods like linear mixed‐effects models. This modelling approach mitigates but does not fully eliminate the impact of missing data and unequal numbers of observations across subjects and time points. Finally, the sample size represents also a limitation: The small cohort size limits statistical power, particularly for diffusions and water T2 metrics. Our findings should therefore be interpreted with appropriate caution and confirmed in larger, independent cohorts.

## 5 Conclusion

Quantitative MRI provides meaningful structural information in LOPD that complements clinical assessments by capturing tissue‐level changes before overt functional decline. FF is the most sensitive imaging marker of progressive muscle involvement, with significant increases detectable prior to measurable changes in clinical outcomes. Water T2 and diffusion metrics showed cross‐sectional associations with functional measures but lacked consistent longitudinal dynamics, indicating unresolved questions about their biological specificity and temporal behaviour in LOPD. Interestingly, higher baseline wT2 values predicted a faster rate of fat replacement, underlining its potential in capturing ongoing pathological processes that precede irreversible tissue loss. Looking forward, integrating qMRI metrics into routine Pompe disease monitoring could bridge the current gap between molecular pathology and clinical function. In contrast to conventional endpoints, which often remain stable for years despite ongoing muscle degeneration, qMRI can reveal tissue‐level alterations with higher sensitivity and objectivity. From a pragmatic standpoint, a minimal MRI dataset should include bilateral fat quantification and wT2 imaging of the thigh. Multicentric harmonisation of acquisition protocols and the establishment of normative datasets will be essential steps toward validating qMRI as a surrogate endpoint for regulatory use.

## Funding

This study was funded by Sanofi (Project number: SGZ‐2019‐12541). JF and AG received grants from the FoRUM program of the Ruhr‐University Bochum (JF: K139‐20, K174N‐22‐A; AG: K‐144‐20). AG is funded by the Heimer Foundation, Bielefeld, Germany. MF is partly financed by the Applied and Engineering Sciences (AES) Dutch Research Council (NWO) (18929). EEK holds an endowed professorship funded by the German Social Accident Insurance (DGUV) since 2020 and has received a grant from the Georg Agricola Ruhr Foundation and DLR e.V.

## Conflicts of Interest

Some authors have received speaker honoraria and travel support from Argenx Germany GmbH, Novartis AG, CSL Behring GmbH, Roche, Alexion and Johnson & Johnson; consultancy fees from Amicus and Roche; and research support from Argenx, Amicus and Alnylam. One author has received travel support from Amicus Therapeutics GmbH and holds stock in Merck KGaA. Additional research funding was provided by the Habilitation Program for Postdoctoral Researchers of the University Hospital Düsseldorf, the Deutsche Gesellschaft für Muskelkranke e.V. and the Deutsche GBS Stiftung e.V. The remaining authors declare no conflicts of interest.

## Supporting information


**FIGURE S1:** Bar plots show mean qMRI metrics for patients with LOPD and control group in muscles with a fat fraction lower than 10%. The lines show the standard deviation. *adjusted *p* < 0.05.

**FIGURE S2:** Longitudinal interaction plots of LOPD patients showing only significant qMRI metric × Time effects between selected qMRI metrics of the thigh and clinical outcomes. Each panel displays: observed individual data points (semitransparent), predicted trajectories from the fixed part of a linear mixed‐effects model (Outcome ~ qMRI metric * Time + (Time Subject)) across the observed predictor range, and a continuous colour gradient encoding the predictor value. Predictions are derived from the model fixed effects (random effects excluded).

**FIGURE S3:** Association between baseline wT2 and change in fat fraction (FF) over the study period. Each point represents one muscle measurement, coloured by subject. The regression line illustrates the positive relationship between baseline wT2 and FF change, adjusted for baseline FF. Higher baseline wT2 values were associated with greater increases in FF (*β* = 0.004 [95% CI: 0.00322; 0.00434], *p* = 0.009).

## Data Availability

The data supporting the findings of this study are available on request from the corresponding author. The data are not publicly available due to privacy or ethical restrictions.
