# Supplementary figures and images for: Longitudinal Assessment of Muscle Involvement in Late‐Onset Pompe Disease Using Quantitative MRI: A Prospective Cohort Study
Source: J Cachexia Sarcopenia Muscle. 2026 Jun 1;17(3):e70304. doi: 10.1002/jcsm.70304 (PMC13239697; doi:10.1002/jcsm.70304)

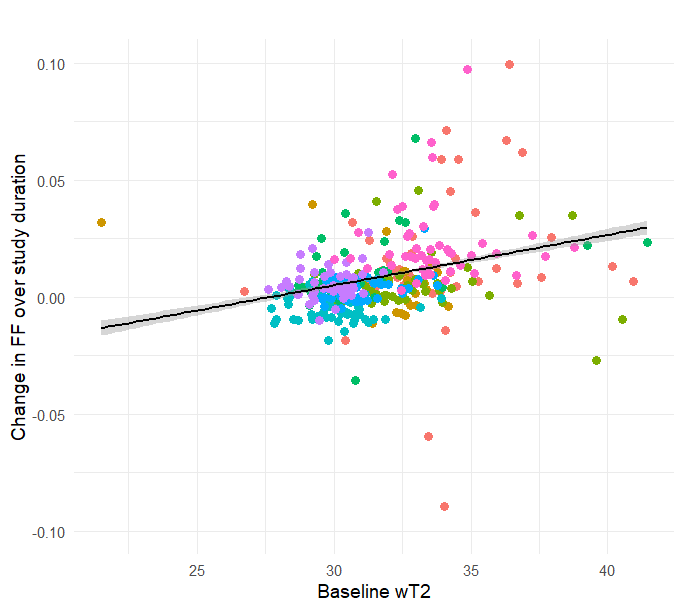

Supplement: Supplementary file 3 — FIGURE S3: Association between baseline wT2 and change in fat fraction (FF) over the study period. Each point represents one muscle measurement, coloured by subject. The regression line illustrates the positive relationship between baseline wT2 and FF change, adjusted for baseline FF. Higher baseline wT2 values were associated with greater increases in FF (β = 0.004 [95% CI: 0.00322; 0.00434], p = 0.009). [file JCSM-17-e70304-s002.tiff]
